# Nickel’s Role in Pancreatic Ductal Adenocarcinoma: Potential Involvement of microRNAs

**DOI:** 10.3390/toxics10030148

**Published:** 2022-03-21

**Authors:** Maria Mortoglou, Luka Manić, Aleksandra Buha Djordjevic, Zorica Bulat, Vladimir Đorđević, Katherine Manis, Elizabeth Valle, Lauren York, David Wallace, Pinar Uysal-Onganer

**Affiliations:** 1 Cancer Research Group, School of Life Sciences, University of Westminster, London W1W 6UW, UK; w1754188@my.westminster.ac.uk; 2 Department of Toxicology “Akademik Danilo Soldatović”, University of Belgrade, 11221 Belgrade, Serbia; lukamanic@outlook.com (L.M.); aleksandra@pharmacy.bg.ac.rs (A.B.D.); zorica.bulat@pharmacy.bg.ac.rs (Z.B.); 3 First Surgical Clinic, Clinical Center of Serbia, 11000 Belgrade, Serbia; vladimir.djordjevic@kcs.ac.rs; 4 College of Osteopathic Medicine, Oklahoma State University Center for Health Sciences, 1111 West 17th Street, Tulsa, OK 74107-1898, USA; kmanis@okstate.edu (K.M.); evalle@okstate.edu (E.V.); lauren.york@okstate.edu (L.Y.); 5 The Department of Pharmacology & Physiology, School of Biomedical Science, Oklahoma State University Center for Health Sciences, 1111 West 17th Street, Tulsa, OK 74107-1898, USA

**Keywords:** pancreatic ductal adenocarcinoma, non-coding RNAs, microRNAs, environmental toxins, nickel, apoptosis

## Abstract

Pancreatic ductal adenocarcinoma (PDAC) is one of the most lethal cancer types with a limited overall survival rate due to the asymptomatic progression of symptoms in metastatic stages of the malignancy and the lack of an early reliable diagnostic biomarker. MicroRNAs (miRs/miRNAs) are small (~18–24 nucleotides), endogenous, non-coding RNAs, which are closely linked to the development of numerous malignancies comprising PDAC. Recent studies have described the role of environmental pollutants such as nickel (Ni) in PDAC, but the mechanisms of Ni-mediated toxicity in cancer are still not completely understood. Specifically, Ni has been found to alter the expression and function of miRs in several malignancies, leading to changes in target gene expression. In this study, we found that levels of Ni were significantly higher in cancerous tissue, thus implicating Ni in pancreatic carcinogenesis. Hence, in vitro studies followed by using both normal and pancreatic tumor cell lines and increasing Ni concentration increased lethality. Comparing LC50 values, Ni-acetate groups demonstrated lower values needed than in NiCl_2_ groups, suggesting greater Ni-acetate. Panc-10.05 cell line appeared the most sensitive to Ni compounds. Exposure to Ni-acetate resulted in an increased phospho-AKT, and decreased FOXO1 expression in Panc-10.05 cells, while NiCl_2_ also increased PTEN expression in Panc-10.05 cells. Specifically, following NiCl_2_ exposure to PDAC cells, the expression levels of miR-221 and miR-155 were significantly upregulated, while the expression levels of miR-126 were significantly decreased. Hence, our study has suggested pilot insights to indicate that the environmental pollutant Ni plays an important role in the progression of PDAC by promoting an association between miRs and Ni exposure during PDAC pathogenesis.

## 1. Introduction

Pancreatic ductal adenocarcinoma (PDAC) is a highly aggressive malignancy, with a 5-year overall survival of less than 8% [1,2]. PDAC patients are symptomless even in the most advanced stages [1]. Systemic chemotherapy with gemcitabine and FOLFIRINOX 5-FU (irinotecan, oxaliplatin, and leucovorin), in combination with radiotherapy is the first-line treatment; however, due to high percentages of toxicity, they present limited efficacy in both elderly and PDAC patients with advanced stages [3,4].

The most common genetic alterations, that are correlated with PDAC prognosis are genetic aberrations in proto-oncogenes including Kirsten rat sarcoma viral homolog (*K-RAS*) and tumour suppressor genes, such as cyclin-dependent kinase inhibitor 2A (*CDKN2A*), tumour protein 53 (*TP53*) and SMAD family number 4 (*SMAD4*) [5,6,7,8]. Recent studies have suggested modifiable risk factors for PDAC such as chronic pancreatitis, diabetes mellitus, and environmental exposures comprising the inhalation of cigarette smoke and exposure to toxic metals, including arsenic, nickel (Ni), and cadmium (Cd) [9,10,11,12].

Ni is a naturally occurring element, which is found in water, soils, air, and sediments, while the International Agency for Research on Cancer (IARC) has classified its compounds as a “group 1” human carcinogen [13,14]. The extensive use of Ni in industrial settings complicates human health effects, with the most scrutinised effect being carcinogenesis [15]. Moreover, the molecular mechanisms of Ni-induced carcinogenesis comprise hypoxia-inducible factor pathways and the generation of oxidative stress, which further lead to DNA damage and the targeting of DNA repair pathways [15,16]. A previous study has suggested a link between Ni content and PDAC [17], while increasing evidence has suggested Ni involvement in several pancreas-related dysfunctions [18].

**Figure 1:**
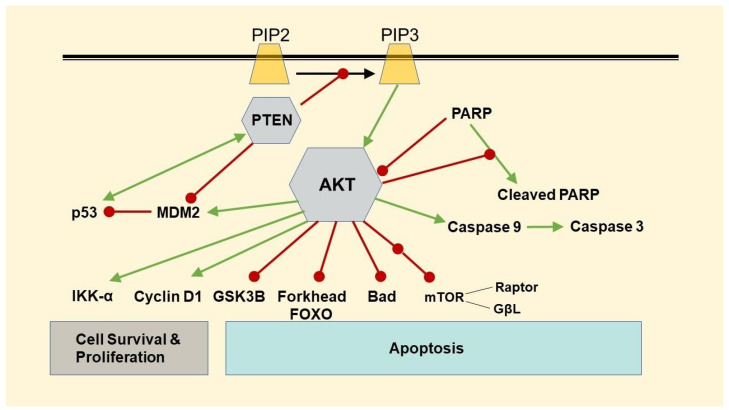
Schematic representing the relationship between proteins associated with apoptosis. Abbreviations: PIP2—phosphatidylinositol 4,5-bisphosphate; PIP3—phosphatidylinositol 3,4,5-trisphosphate; PTEN—phosphatase and tensin homolog; AKT—Ak strain transforming; GSK3β—glycogen synthase kinase 3 beta; IKK-ɑ—IkappaB kinase; MDM2—mouse double minute 2 homolog or E3 ubiquitin-protein ligase; PARP—poly (ADP-ribose) polymerase; mTOR—mechanistic target of rapamycin; Bad—Bcl-2-associated death promoter. Green arrows represent stimulation and red arrows represent inhibition.

The activity of AKT (protein kinase B) plays a pivotal role in regulating multiple systems involved in cellular growth (glucose metabolism, cell proliferation, transcription, cell migration) and apoptosis (Figure 1). Using this potentially critical role for AKT in PDAC [19,20,21,22], we chose to examine the overall lethality of NiCl_2_ and Ni-acetate. By performing LC50 analysis, we determined concentrations that are subtoxic and could elicit molecular changes before cell death for future studies. We then selected phospho-AKT (p-AKT), PTEN, p53 (total), PARP, FOXO1, β-catenin, and cleaved caspase-3 protein expression, caspase 3/7 as our markers for Ni-related toxicity. Each of these proteins is involved in the progression of PDAC. The question remains: are these proteins direct regulators of PDAC progression, or does dysregulation up- or downstream alter protein function?

The loss of PTEN function has been reported as an underlying cause of PDAC development [23,24]. The loss of PTEN function removes the inhibitor input on AKT, thus increasing AKT activity, leading to tumour development. Numerous investigators have reported a loss of p53 function via a loss or mutation of the protein in PDAC tissue, implicating this loss of function as an underlying cause of cell migration and metastasis [25,26]. Ni compounds have been implicated in an increase in oxidative stress. The overactivity of PARP has been suggested to play an important role in PDAC progression following oxidative stress, which promotes cell necrosis and pro-inflammatory gene expression, contributing to cancer development. PARP inhibitors are being investigated as potential anti-cancer therapies, and evidence suggests that mutated PARP may be a factor in the development of BRCA-mutant PDAC [27,28]. PARP inhibitors may have utility through AKT activation, as well as increased apoptosis and mitochondrial protection during oxidative stress [29]. The β-catenin/FOXO system activity is enhanced in the presence of free radicals [30]. Elevated oxidative stress has been implicated in PDAC development, and the relationship between FOXO1 activity and the Wnt/β-catenin system is critical for maintaining normal cellular function. The cellular loss of FOXO1 results in the presentation of stem-like cells that promote the development of PDAC [31,32]. The FOXO1-mediated inhibition of PDAC formation is partially due to the inhibition of β-catenin activity, which promotes tumour formation [33]. FOXO1 activity is negatively moderated by miRs (miR-21 and miR-27a), which are upregulated in PDAC. Increased miR-21 and miR-27a activity decreases FOXO1 expression/activity, effectively preventing FOXO1’s control of β-catenin [34,35]. The regulation of proteins associated with cellular function, growth, and proliferation is complex and subject to multiple regulatory influences. Therefore, in this study, we underline the foundation of the mechanistic effects of Ni on the cellular function of PDAC by examining different proteins involved in either apoptosis, cell proliferation, or both (Figure 1).

The microRNAs (miRs) are small, non-coding, evolutionarily conserved, single-stranded RNA molecules, which can regulate gene expression at the post-transcriptional level [36]. These miRs are involved in several biological processes, including metastasis, apoptosis, angiogenesis, invasion, and proliferation, and they are reported to moderate several cell signalling pathways related to various malignancies, including PDAC [7,8,12,37,38,39,40,41,42,43,44,45]. Aberrant expression levels of miRs are closely linked to PDAC pathogenesis [7,8,41,46].

The upregulation of miR-221 is commonly observed in PDAC tissue samples, with a diagnostic specificity of 93% [47]. The overexpression of miR-221 has been associated with several other malignancies, including hepatocellular carcinoma, as well as prostate and colorectal cancers [48,49,50,51]. Furthermore, increased expression levels of miR-155 were reported in PDAC tissue samples compared with healthy controls [52]. The upregulation of miR-155 is correlated with poor survival and prognosis in PDAC patients, and pancreatic intraepithelial neoplasia 2 (PanIN-2) to PanIN-3 [7,8,53,54]. The miR-126 is an example of the tumour suppressor miR, which has been associated with lung, gastric, breast, and prostate cancers, as well as PDAC [44,55,56,57]. In particularl, in PDAC, miR-126 dysregulation is due to the post-transcriptional upregulation of *KRAS* [58] and *HER2* [59], which is commonly present in PDAC cases.

Our previous studies have shown that environmental toxins, such as Cd, can promote their toxicity through miRs [12,18,60]. Toxic metals such as Ni have not been studied as extensively as Cd, and, therefore, only a small number of miRs associated with Ni exposure has been characterized. The functions and mechanisms of these miRs in carcinogenesis remain to be elucidated. Therefore, our aims were to detect Ni concentrations in both human tumour and non-tumour tissue samples, assess NiCl_2_- and Ni-acetate-mediated toxicity during PDAC progression, and investigate the role of miRs in response to NiCl_2_ exposure. In the current study, a newly found role of miRs was studied in Ni-induced PDAC carcinogenesis, and the cellular and molecular changes in the control and PDAC cell lines following exposure to NiCl_2_ or Ni-acetate were also examined.

## 2. Materials and Methods

### 2.1. Study Population and Sampling

A case-control study was conducted at the First Surgical Clinic, University Clinical Center of Serbia, and the Faculty of Pharmacy, University of Belgrade. Twenty-eight patients with histopathologically confirmed exocrine pancreatic carcinoma were involved in the study. No exclusion criteria regarding the age, sex, or cancer stage were set. All patients were citizens of the Republic of Serbia and were enrolled in the study between May 2014 and December 2016, prior to receiving any chemotherapy or radiotherapy. After the study aim, procedures, and risks were explained to the patients, each enrolled patient confirmed they understood everything and consented to participate in the study. Samples of the cancerous tissue (approximately 1 g each) were obtained by excision during Whipple procedure or total pancreatectomy at the First Surgical Clinic, University Clinical Center of Serbia. Twenty samples were taken as controls during routine post-mortem examination of twenty patients whose death did not occur due to cancer at the Department of Forensic Medicine, Faculty of Medicine, University of Belgrade. A Forensic Medicine Specialist confirmed the absence of malignancies in control samples. Fresh, untreated control samples were frozen and kept at −20 °C. In this way, the test and control and cancer samples were prepared and analyzed identically. The study was approved by the Ethics Committee of the University Clinical Center of Serbia (Approval No. 31/8).

### 2.2. Human Sample Analysis

The exact mass of the samples was determined prior to mineralization. A mix of 7 mL p.a. nitric acid (65% W/V, Merck, Darmstat, Germany) and 1 mL p.a. hydrogen peroxide (30%, W/V, Sigma-Aldrich, Saint Louis, MI, USA) was used for mineralization in a microwave oven (Milestone Ethos One, Milestone, Sorisole, Italy) with SK-10 Teflon rotor and cuvettes rinsed with nitric acid thrice (Milestone, Sorisole, Italy). The samples were heated to 180 °C for 15 min (at 1000 W and 50 bar), then digested for 15 min (at 180 °C, 1000 W, and 50 bar), and cooled to room temperature over 15 min. Mineralized samples were transferred quantitatively to 25-millilitre volumetric flasks and diluted using redistilled water. Ni concentration was determined in the obtained solutions. The blank was prepared by following the same steps, only without a tissue sample. Ni concentration was determined using Agilent 200 Series AA AAS (Agilent Technologies, Santa Clara, CA, USA), using an Agilent AAS GTA120 graphite cuvette (Agilent Technologies, USA) against the blank. A series of standard solutions was prepared by diluting 1000 mg/mL nickel (II) nitrate solution (Merck, Darmstat, Germany) with redistilled water.

### 2.3. Pancreatic Cell Cultures and Nickel Treatment

Cell lines were purchased from the American Type Culture Collection (ATCC, Manassas, VA, USA). Control pancreas cells (hTERT-HPNE (“human pancreatic Nestin-expressing” cells or HPNE; ATCC^®^ CRL-4023™, control pancreatic cells)) and tumour (AsPC-1 (ATCC^®^ CRL-1682™, pancreatic tumour cells), Panc-1 (ATCC #CRL-1469™, Pancreas ductal epithelioid carcinoma), Panc-10.05 (ATCC #CRL-2547™, Pancreatic epithelial adenocarcinoma), MiaPaCa-2 (ATCC#CRL-1420™, Pancreas ductal epithelioid carcinoma), and BxPC-3 (ATCC #CRL-1687™, Pancreatic adenocarcinoma)) were cultured according to the ATCC-suggested protocols. Unless otherwise specified, cells were grown in their defined optimum growth media, as defined by ATCC. For a parallel set of LC50 assays, cells were grown in a minimal media of MEM plus 1% foetal bovine serum (FBS). In miR studies, cells were cultured and treated with NiCl_2_ (NiCl_2_; 50 μM), as described in our previously published data [11].

### 2.4. Chemicals and Antibodies

Media for each cell line was purchased from Corning Life Sciences (Tewksbury, MA, USA). Media supplements were obtained from Sigma-Aldrich (St. Louis, MO, USA), including penicillin/streptomycin, glucose, glutamine, sodium bicarbonate. FBS, triple-0.1-micrometer-filtered, was purchased from Atlanta Biologicals through R&D Systems (Minneapolis, MN, USA). NiCl_2_, Ni-acetate, sterile dimethylsulfoxide (DMSO). Sterile phosphate-buffered saline (PBS) and molecular-grade water were purchased from Sigma-Aldrich (St. Louis, MO, USA), Fisher Scientific (Houston, TX, USA), and Pierce Biotechnology (Rockford, IL, USA). ELISA kits were obtained from Pierce Biotechnology (Rockford, IL, USA), while antibodies were purchased from R&D Systems Inc., (Minneapolis, MN, USA), Cell Signaling Technology, Inc. (Danvers, MA, USA), and Thermo Fisher Scientific (Waltham, MA, USA).

### 2.5. LC50 Assays to Determine Nickel Toxicity after 48 h of Exposure

Each of the five cell lines was plated as it has been previously described [60]. Cells were incubated for 48 h with increasing concentrations of the Ni compounds or vehicle (12 concentrations, 0–1 mM). After exposure, an 11-millimolar stock MTT (3-(4,5-dimethylthiazol-2-yl)-2,5-diphenyltetrazolium bromide) stock solution was prepared in PBS (1.1 mM of final concentration in each well) and added to each well. Plates were returned to the incubator (37 °C/5% CO_2_) for 4 h. A total of 50 μL of DMSO was added to each well to solubilize the formazan crystals and the plate incubated for an additional 30 min at 37 °C. Viable cells were then measured by the absorbance at 540 nm (BioTek-HT plate reader, Agilent, Santa Clara, CA, USA), which is directly proportional to the number of live cells.

### 2.6. Changes in Protein Expression

The measurement of β-catenin was determined using a commercially available kit for measuring human β-catenin in cell lysates (MyBioSource, Inc. #MBS266009; San Diego, CA, USA) and used as it has described in our previously published data based on the manufacturer’s protocol [60]. β-catenin was quantified by measuring the absorbance at 450 nm and extrapolating to the standard curve.

ELISAs (Pierce Biotechnology, Rockford, IL, USA) were performed using the methods described in the assay kit and each of the assays was performed under identical conditions, with the exception of antibody dilutions (phospho-Akt (AF887, R&D Systems; 1:2000); PTEN (AF847, R&D Systems; 1:1000); PARP (1861790, Thermo-Fisher; 1:1000); FOXO1 (2880S, Cell Signaling; 1:1000); p53 (1861777, Thermo-Fisher; 1:1000) and cleaved (Asp175) cleaved caspase 3 (PA5-114687, Thermo-Fisher Invitrogen; 1:1000)). Cells were exposed to 50 μM NiCl_2_, Ni-acetate or vehicle for 48 h in a 37 °C/5% CO_2_ incubator (ThermoFisher #3120, Waltham, MA, USA). Following exposure, 4% formaldehyde (Pierce Biotechnology, Rockford, IL, USA) was added to fix the cells. Cells were prepared according to manufacturer’s guidelines; the primary antibody was added and the plates sealed with microplate sealing film (Fisher Scientific, Houston, TX) and stored overnight at +4 °C. Excess antibody was removed by washing, followed by the addition of TMB (3,3′, 5,5-tetramethylbenzidine). Absorbance was measured at 450 nm and values were standardized to cell number using Janus Green whole-cell stain (Sigma-Aldrich, St. Louis, MO). After staining with Janus Green, the absorbance was measured at 615 nm. Dividing the absorbance at 450 nm by the absorbance at 615 nm yielded the relative protein expression standardized to live cell number.

### 2.7. Caspase 3/7 Kinetic Assays

Caspase 3/7 activity (measurement of apoptosis) was measured by using the commercially available kit caspase 3/7 conversion of the non-fluorescent substrate Z-DEVD-R110 bis-(N-CBZL-aspartyl-L-glutamyl-L-valyl-L-aspartic acid amide;) to the rhodamine 110 leaving group, which fluoresces at 499 nm_ex_/521 nm_em_ (Apo-One™ Homogeneous caspase-3/7 assay, Promega, Madison, WI, USA). The fluorescence generated was directly proportional to the amount of caspase 3 and 7 present. Cells were maintained and plated as described above. Exposure was initiated by the addition of 50 μM NiCl_2_, Ni-acetate or vehicle for 48 h in a 37 °C/5% CO_2_ incubator. After exposure, caspase 3/7 activity was determined as it has described previously [60]. Emitted fluorescence was measured using a Bio-Tek^®^ plate reader and Gen5 software at 485_ex_/530_em_.

### 2.8. RNA Extraction and Analysis of miR Expression

The miR expression levels were examined in control (non-treated) and treated cells with NiCl_2_. Panc-1 and MiaPaCa-2 PDAC cell lines. RNA extraction was carried out using Trizol (Sigma, Hertfordshire, UK). Reverse transcription of RNA to cDNA was carried out using a miRCURYâLNAâ RT Kit (Qiagen, Manchester, UK). The miRCURYâ LNAâ miRNA SYBRâ Green (Qiagen, Manchester, UK) was used in conjunction with MystiCq microRNA qPCR primers for miR-155 (hsa-miR-155-5p), miR-126 (hsa-miR-126-5p), and miR-221 (hsa-miR-221-5p), which were all obtained from Sigma (Paisley, UK). RNU6-snRNA was used as reference RNA to normalise miR expression levels, forward, 5′-GCTTCGGCAGCACATATACTAAAAT-3′ and reverse 5′-CGCTTCACGAATTTGCGTGTCAT-3. The conditions for thermocycling were as described previously [60]. The miR-155, miR-126, and miR-221 expression levels were normalised to RNU6 using the 2^^ΔΔCT^ method [61].

### 2.9. Statistical Analysis

IBM SPSS (v18.0, IBM Corporation, Armonk, NY, USA) was used to analyse Ni tissue content. Ni concentration was determined from a standard curve; subsequently, Ni content in fresh tissue samples in ng/g was calculated. Kolmogorov–Smirnov test was used to test data normality, then the data were analysed using Mann–Whitney U-test (*p* < 0.05 was considered significant). GraphPad Prism version 9.03 (GraphPad Software, San Diego, CA, USA) was used for other statistical analyses and preparation of graphs. t-test was utilized together with Tukey’s post hoc analysis to examine miRs expression levels in response to NiCl_2_ treatment. Experiments were carried out in triplicates for miRs analysis. miR-related data were analysed using GraphPad Prism (v 9.1-9.3; San Diego, CA). Analyses included: (1) one-way ANOVA with the specialised Dunnett’s test for post hoc comparison to control values, (2) two-way (cell type x treatment) or three-way (cell type x treatment x protein) ANOVA with Tukey’s test adjusted for multiple comparisons or Sidak’s post hoc comparison test. The performed analyses are listed in the results section, or the appropriate figure legend. LC50 values were determined by fitting the data to a nonlinear curve fitting, log concentration x response (three-parameter) single-site model, to yield a sigmoid inhibition curve. All data, except for Ni content assays, are expressed as the mean ± SD of 4–8 assays performed in duplicate or triplicate where indicated. For data that violated the assumption of normal distribution, the nonparametric Kruskal-Wallis test was performed, followed by a post hoc comparison using the corrected Dunn’s test. Significance is set at α = 0.05.

## 3. Results

### 3.1. Ni Concentration in PDAC Tissue Samples

**Figure 2:**
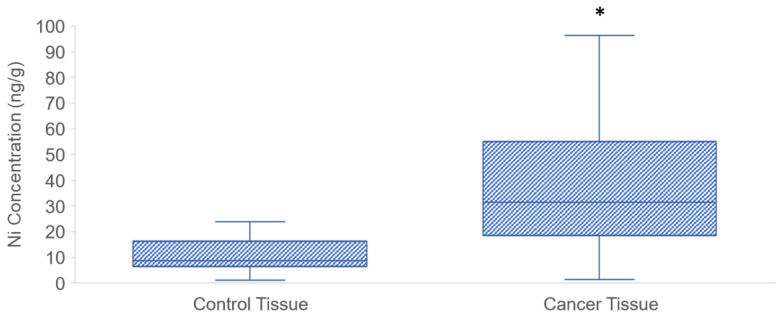
Levels of Ni in human pancreatic tissue in the investigated population. Boxes represent interquartile intervals (25–75%), while lines inside boxes represent medians. The box represents the interquartile range (25–75th percentile), the line within the box represents median value and ends of the whiskers represent the minimum and maximum values within the group. The asterisk indicates a statistically significant difference from the control levels (Mann–Whitney U test, *p* < 0.001).

In total, 28 patients with histological PDAC diagnosis (cases) and 20 death cases unrelated to cancer (controls) showed Ni levels. After the tissue mineralization, graphite furnace atomic absorption spectrometry was used to determine the Ni concentrations. As shown in Figure 2, the Ni levels in the cancerous tissue (1.46–192.10 ng Ni/g wet tissue) were significantly higher than the control levels (1.22–24.06 ng Ni/g). According to the Kolmogorov-Smirnov test, median values were used since the collected data did not follow a normal distribution. Median Ni concentration in control tissue was 8.88 ng/g. Median Ni concentration in cancerous tissue was 31.54 ng/g and was statistically significantly higher than in control tissue (*p* < 0.01).

### 3.2. Determination of LC50 Values for NiCl_2_ and Ni-Acetate in PDAC Cell Lines

**Figure 3:**
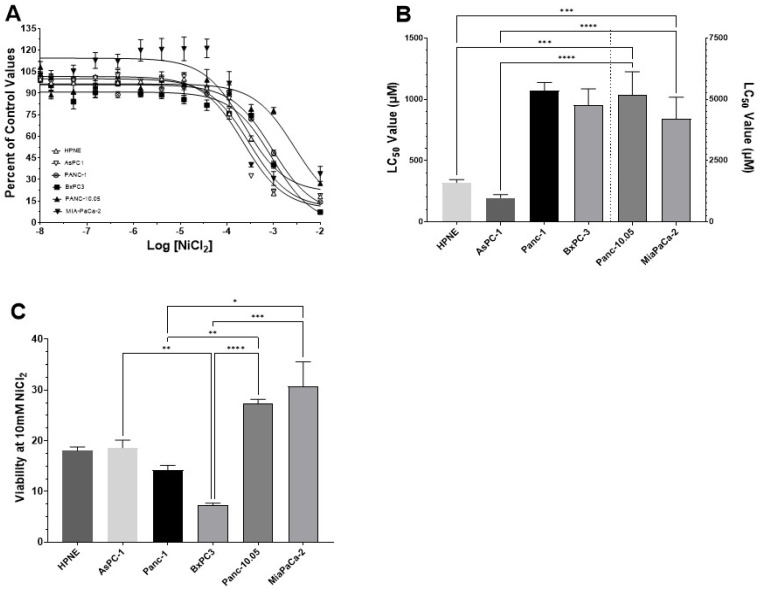
LC50 curves for NiCl_2_ in PDAC cell lines. Cells were exposed to increasing concentrations of NiCl_2_ (0–10 mM) for 48 h and cell viability was measured using the MTT assay. Curve shapes best fitted a single site model (three-parameter) (**A**). The rank order for NiCl_2_ LC50 values was AsPC-1 < HPNE < BxPC-3 = Panc-1 << Panc-10.05 = MiaPaCa-2 (**B**). Comparing lethality at 10 mM (**C**), with viability expressed as percent of control values, the BxPC-3 was the most sensitive cell line, whereas the Panc-10.05 and MiaPaCa-2 cells were the least sensitive. Data are expressed as the mean ±SD of eight assays performed in duplicate (* *p* < 0.05; ** *p* < 0.01; *** *p* < 0.001; **** *p* < 0.0001).

**Figure 4:**
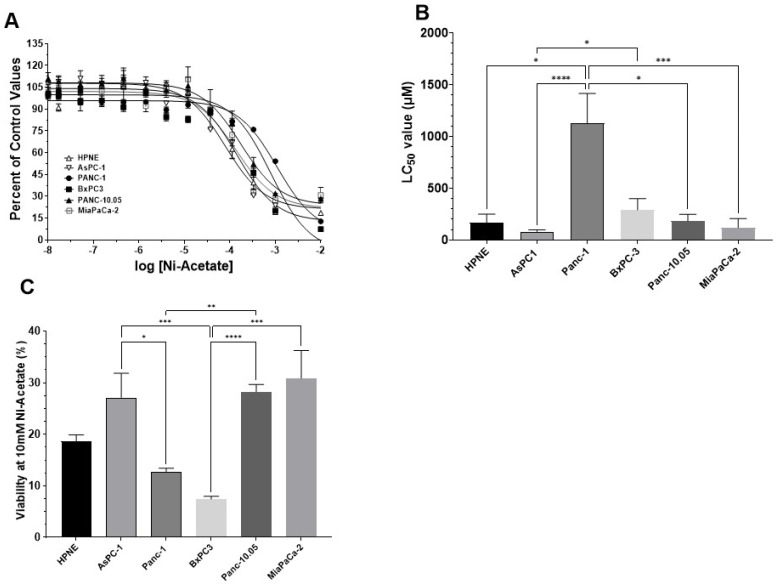
LC50 curves for Ni-acetate in PDAC cell lines. Cells were exposed to increasing concentrations of NiCl_2_ (0–10 mM) for 48 h and cell viability was measured using the MTT assay. Curve shapes were best fitted to a single site model (three-parameter) (**A**). The rank order for Ni-acetate LC50 values was AsPC-1 < MiaPaCa-2 ≤ HPNE ≤ Panc-10.05 < BxPC-3 << Panc-1. (**B**). Except for the Panc-1 cells, all the PDAC cell lines exhibited lower LC50 values after exposure to Ni-acetate compared to NiCl_2_. Comparing lethality at 10 mM (**C**), the BxPC-3 was the most sensitive of the cell lines whereas AsPC-1 and Panc-10.05 cells were least sensitive. Data are expressed as the mean ± SD of eight assays performed in duplicate (* *p* < 0.05; ** *p* < 0.01; *** *p* < 0.001; **** *p* < 0.0001).

We determined the LC50 values for NiCl_2_ and Ni-acetate in multiple PDAC cell lines. The initial LC50 analysis established baseline values for subsequent experimentation. Each cell line was incubated in its optimum growth media and exposure started with the addition of increasing concentrations of NiCl_2_ (Figure 3A–C) or Ni-acetate (Figure 4A–C). The plates were returned to the incubator (37 °C/5% CO_2_) for 48 h. There was a reduction in cell viability with increasing concentrations of either NiCl_2_ (Figure 3A) or Ni-acetate (Figure 4A). The shapes of the curves were best fitted to a single site model (three parameter model). When examining the curves, the HPNE and AsPC-1 cell lines were shifted to the left (lower LC50), followed by the Panc-1 and BxPC-3 cells, with the Panc-10.05 and MiaPaCa-2 cells exhibiting LC50 values to NiCl_2_ of >5000 μM and 4209 μM, respectively (Figure 3A). The rank order for NiCl_2_ LC50 values was AsPC-1 < HPNE < BxPC-3 = Panc-1 << Panc-10.05 = MiaPaCa-2. These differences in LC50 values are reflected in Figure 3B and Table 1. For NiCl_2_ exposure, the Kruskal–Wallis analysis revealed a significant difference in LC50 values between cell lines H(6) = 41.64; *p* < 0.0001. The differences between the groups were determined with Dunn’s test for multiple comparisons, as shown in Figure 3B and Table 1. The maximum lethality, at 10 mM NiCl_2_, demonstrated a significant effect across the cell lines H(6) = 37.72; *p* < 0.0001 (Figure 3C). Interestingly, BxPC-3 appeared to be the most sensitive cell line, with <10% viability at 10 mM NiCl_2_. The Panc-10.05 and MiaPaCa-2 cells appeared to have the greatest resistance to NiCl_2_ toxicity. with nearly 25–30% cell viability at 10 mM NiCl_2_. The HPNE control cells exhibited only modest (19%) resistance to the toxicity of NiCl_2_.

There was a reduction in cell viability with increasing concentrations of Ni-acetate (Figure 4A). The shapes of the curves were best fitted to a single site model (three parameter model). The chemical form of Ni appeared to have a robust effect with the LC50 for all the cell lines, except Panc-1, which was significantly reduced when exposed to Ni-acetate compared to the LC50 values for NiCl_2_. The largest reduction was observed in the anc-10.05 cells, which changed from >5000 μM to 183.5 μM, followed by the MiaPaCa-2 cells, which had an LC50 value for NiCl_2_ in excess of 4200 μM to 119 μM for Ni-acetate. The potency series following Ni-acetate exposure was AsPC-1 < MiaPaCa-2 ≤ HPNE ≤ Panc-10.05 < BxPC-3 << Panc-1. These differences in LC50 values are reflected in Figure 4B and Table 1. For Ni-acetate exposure, the Kruskal–Wallis analysis revealed a significant difference in LC50 values between cell lines H(6) = 32.66; *p* < 0.0001. The differences between the groups were determined with Dunn’s test for multiple comparisons and are shown in both Figure 4B and Table 1. The maximum lethality at 10 mM Ni-acetate was determined and we observed a significant effect across the cell lines H(6= 37.27; *p* < 0.0001 (Figure 4C). Similar to our observation following NiCl_2_ exposure, the BxPC-3 cell line was the most sensitive to the toxic effects of Ni-acetate, with < 10% viability at 10 mM N-acetate. The Panc-10.05, MiaPaCa-2, and AsPC-1 cells appeared to have the greatest resistance to Ni-acetate toxicity, with approximately 25–30% cell viability at 10 mM Ni-acetate. The HPNE control cells exhibited only modest (19%) resistance to Ni-acetate toxicity, which was similar to the outcome after exposure to NiCl_2_.

### 3.3. Changes in Protein Expressions of β-Catenin, Phospho-AKT, p53, and FOXO-1

**Figure 5:**
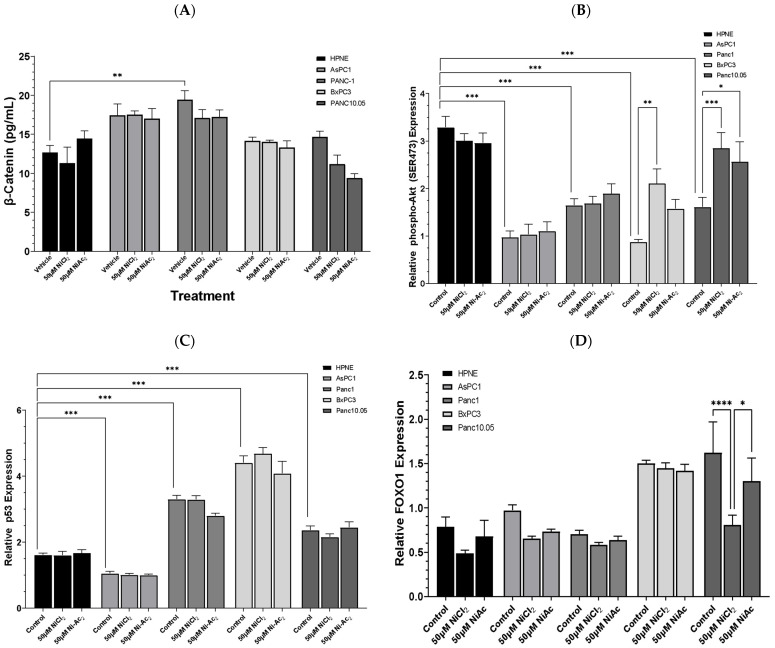
Quantification and expression of key cell function proteins after 48 h exposure to 50 μM NiCl_2_ or Ni-acetate in PDAC cell lines. These assays quantified the amount of β-catenin (**A**), phospho-AKT (**B**), p53 (**C**), and FOXO-1 (**D**). The predominant difference was in the basal levels of each protein and the differences between the cell lines. The expressions of phospho-AKT (**B**) and p53 (**C**) typify this response. Treatment with either NiCl_2_ or Ni-acetate had little effect after 48 h, except for phospho-AKT (**B**) and FOXO-1 (**D**), in which NiCl_2_ and Ni-acetate increased phospho-AKT expression and decreased FOXO-1 expression in Panc-10.05 cells. In BxPC-3 cells, exposure to NiCl_2_ increased phospho-AKT expression 146% compared to vehicle control. Data are expressed as the mean ± SD of 4 assays performed in duplicate (* *p* < 0.05; ** *p* < 0.01; *** *p* < 0.001; **** *p* < 0.0001).

We then examined the mechanistic effects of Ni on the cellular function of PDAC by assessing key proteins. Briefly, the cells were exposed to 50 μM NiCl_2_ or Ni-acetate for 48 h and the quantity of β-catenin was measured using a whole-human-cell assay kit (Figure 5A). Exposure to the Ni compounds did not result in robust changes in β-catenin content in any of the cell lines, although there was a slight downward trend in the Panc-10.05 cell line. The quantity of β-catenin was significantly influenced by the cell line (F_4,30_ = 20.37; *p* < 0.0001). The basal content (control) of β-catenin was approximately 40% higher in the Panc-1 cells compared to the HPNE control cells (*p* < 0.01). ELISAs were used to determine the protein content in the PDAC cell lines. The measurement of relative phospho-AKT is presented in Figure 5B. There were significant differences between the cell lines (F_4,45_ = 37.27; *p* < 0.0001), as well as a significant effect of treatment (F_2,45_ = 5.57; *p* = 0.0069) and a significant interaction between cell line and treatment (F_8,45_ = 2.833; *p* = 0.012). The HPNE cells demonstrated minimal changes following NiCl_2_ or Ni-acetate exposure, but the basal content of phospho-AKT was nearly 50% higher than in the tumour cell lines (*p* < 0.001). The only cell lines in which an effect of the Ni compounds was observed were the BxPC-3 and Panc-10.05 cells, where NiCl_2_ exposure significantly elevated the expression of phospho-AKT. The expression of p53 (Figure 5C) displayed a similar pattern to β-catenin. There was a significant effect based on the cell line (F_4,45_ = 239.0; *p* < 0.0001). There were no treatment-related effects, but the basal level of p53 expression was up to twofold higher in the Panc-1, BxPC-3, and Panc-10.05 cells compared to the HPNE cells (*p* < 0.001). The AsPC-1 cells exhibited a 30% reduction in p53 expression compared to the HPNE cells (*p* < 0.001). The expression of FOXO-1 continued the trend of cell-line-mediated differences (F_4,43_ = 32.64; *p* < 0.0001), with the BxPC-3 and Panc10.05 cells expressing the highest content of FOXO-1. There was a significant effect of treatment (F_2,43_ = 10.14; *p* = 0.0002), with the Panc-10.05 cells exhibiting the greatest sensitivity to the effects of NiCl_2_ through a 50% reduction in FOXO-1 expression compared to the Panc-10.05 vehicle control (*p* < 0.0001). Exposure to Ni-acetate resulted in a blunted reduction in FOXO-1 expression by 19% compared to the Panc-10.05 vehicle control (*p* < 0.05).

### 3.4. Changes in Protein Expressions of PTEN and PARP, and Caspase 3/7 Activity

**Figure 6:**
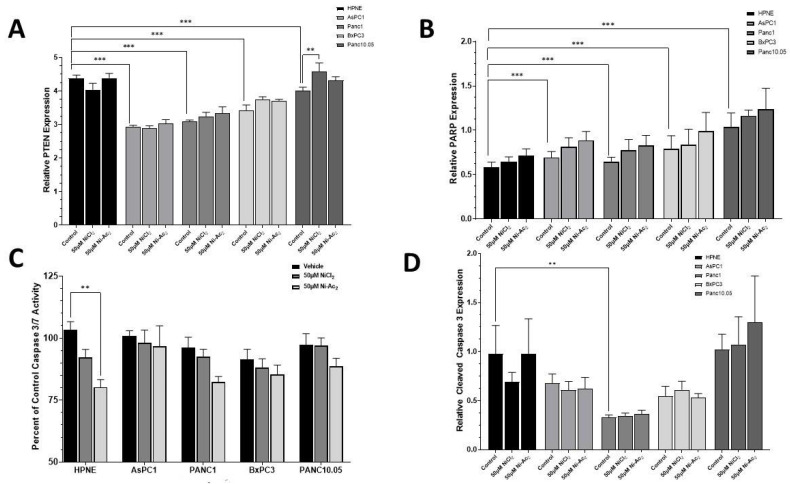
Quantification and expression of key cell-function proteins after 48 h exposure to 50 μM NiCl_2_ or Ni-acetate in PDAC cell lines. These assays quantified the amount of PTEN (**A**), PARP (**B**), caspase 3/7 activity (**C**), and cleaved (active) caspase 3 expression (**D**). The major difference observed between groups was dependent on the cell line, with each of the PDAC cell lines expressing different protein levels. Treatment effects were evident in PTEN expression in the Panc-10.05 cell line, in which NiCl_2_ exposure resulted in a significant (*p* < 0.01) increase in PTEN expression. Caspase 3/7 activity was significantly (*p* < 0.01) reduced after exposure to Ni-acetate. Data are expressed as the mean ±SD of four (protein expression) or eight (caspase 3/7 activity) assays performed in duplicate (** *p* < 0.01; *** *p* < 0.001).

The cells were incubated with 50 μM NiCl_2_ or Ni-acetate for 48 h, and the expression of PTEN (Figure 6A), PARP (Figure 6B) was quantified. The PTEN expression was dependent on the cell line (F_4,45_ = 65.3; *p* < 0.0001), with higher levels of basal expression in the HPNE control group compared to each of the PDAC cell-line controls (*p* < 0.001). Exposure to 50 μM NiCl_2_ significantly increased the PTEN expression in the Panc10.05 cells compared to their vehicle control (*p* < 0.01). The expression of PARP exhibited an inverse profile compared to what was observed with the PTEN expression (F_4,45_ = 5.33; *p* = 0.004). The HPNE control cells had significantly lower PARP expression than each PDAC cell-line control group (*p* < 0.001). The treatment significantly impacted the activity of caspase (Figure 6C), as did the cell line (F_4,105_ = 2.79; *p* = 0.0299) with a trend towards reductions in caspase activity (F_2,105_ = 9.65; *p* = 0.0001). Exposure to NiCl_2_ resulted in small, non-significant, reductions in caspase 3/7 activity. Exposure to Ni-acetate resulted in larger reductions in caspase 3/7 activity, with a significant (*p* < 0.01) 20.6% reduction in the HPNE group. The expression of cleaved (active) caspase 3 (Figure 6D) was significantly (F_4,45_ = 27.79; *p* < 0.0001) dependent on the cell line, with a minimal effect of treatment. Only the Panc-1 control group exhibited significantly (*p* < 0.01) lower expression than the HPNE control group.

### 3.5. Changes in miR Expression Levels in Response to Nickel Chloride Treatments in PDAC Cells

**Figure 7:**
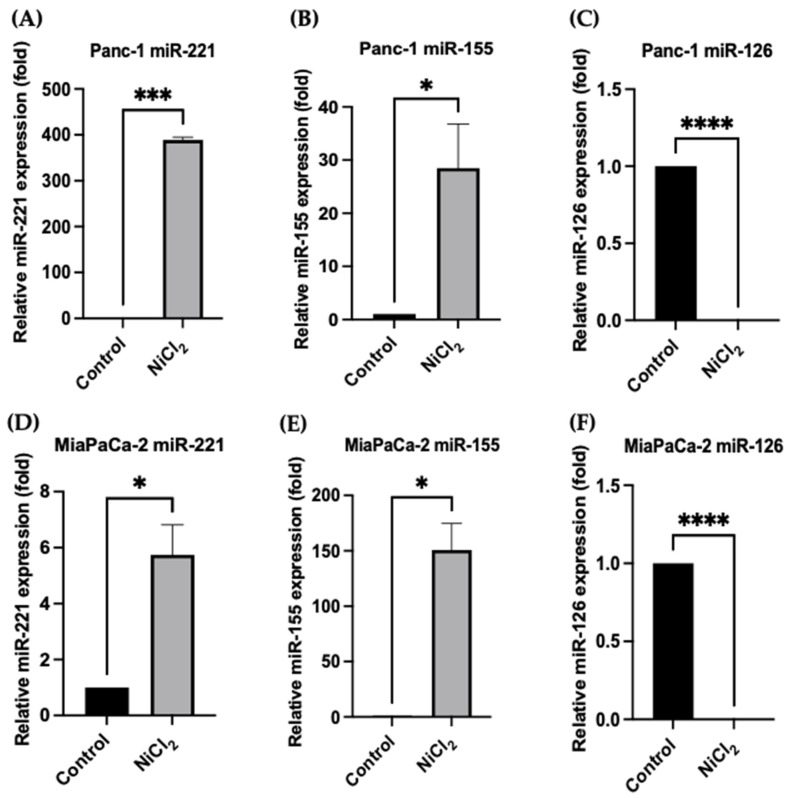
NiCl_2_ treatment-mediated effects on miR expression levels in PDAC cells. (**A**–**C**): Effects of NiCl_2_ in the Panc-1 cell line: miR-221 relative expression levels (**A**); miR-155 relative expression levels (**B**); miR-126 relative expression levels (**C**). (**D**–**F**): Effects of NiCl_2_ in the MiaPaCa-2 cell line: miR-221 relative expression levels (**D**); miR-155 relative expression levels (**E**); miR-126 relative expression levels (**F**). NiCl_2_ treatment effects were evident in the expression levels of miR-221 (*p* < 0.001; Panc-1, *p* < 0.05; MiaPaCa-2), and miR-155 (*p* < 0.05 for all) compared to the control cells, while the expression levels of miR-126 were significantly downregulated (*p* < 0.0001 for all). The column graphic represents the average of three replicates of RNA isolated from the Panc-1 and MiaPaCa-2 cell lines. Data normalized according to RNU6 expression by fold analysis (n = 3, *p* < 0.05); exact *p*-values are indicated (* *p* ≤ 0.05; *** *p* ≤ 0.001; **** *p* ≤ 0.0001); error bars indicate SD.

After investigating the mode of action of NiCl_2_ in apoptosis, we further analysed its effect on miR expression by using the Panc-1 and MiaPaCa-2 PDAC cell lines as a model, based on our previous studies [41,60]. The miR-221, miR-155, and miR-126 are closely linked to PDAC progression in different stages [41,54,62,63]. Following incubation, NiCl_2_, miR-221 expression was elevated both in Panc-1 and MiaPaCa-2 cells (385-fold, *p* < 0.001, and 5.8-fold, *p* < 0.05, respectively; Figure 7A,D; n = 3 for both). Similarly, an upregulation of miR-155 was noted in both the Panc-1 (28.5-fold) and MiaPaCa-2 (151-fold) cells after NiCl_2_ exposure (Figure 7B,E; n = 3, *p* < 0.05 for both), while NiCl_2_ treatment reduced miR-126 expression considerably in Panc-1 (96.6-fold) and MiaPaCa-2 (100-fold) (Figure 7C,F; n = 3, *p* < 0.0001 for both). Our results suggest that NiCl_2_, increases oncogenic miR expressions, such as miR-221 and miR-155, while reducing the expression levels of the tumour suppressor, miR-126. To date, this is the first analysis to show that NiCl_2_ could influence the expression levels of these miRs in PDAC.

## 4. Discussion

The primary results of this study are that (1) a positive correlation between Ni concentration and the incidence of PDAC was found in the PDAC tissue samples; (2) exposure to Ni was toxic to all the PDAC cells and, although Ni-acetate appeared to be most toxic, both NiCl_2_ and Ni-acetate alter the expression of phospho-AKT, PTEN, and FOXO-1; (3) the oncogenic miR-221, miR-155, and tumour suppressor miR-126 were highly dysregulated in the PDAC cells treated with NiCl_2_ compared with the control PDAC cells.

Ni can stimulate global hypermethylation, which results in the suppression of key tumour-suppressor genes, the activation of proto-oncogenes, and senescence [64]. Oxidative stress is another important route for Ni-induced carcinogenesis [65], while the hypoxia-inducible signaling pathway is a key mechanical route for Ni-induced carcinogenesis, as the transcription factor hypoxia-inducible-factor-1 (HIF-1) is triggered [66].

Our current study provides some pilot insights into Ni concentration and incidence of PDAC based on in vivo data. Ni concentrations determined in this study are comparable to those observed in other studies. Namely, Ni concentration determined in organ samples obtained post-mortem from subjects without pancreatic cancer were ranging from 1 to 19 ng/g [67]. Higher Ni content in tissue samples obtained from cancer patients has been observed in other studies. Depending on the biological material sampled, conflicting results have been published, mainly relying on clipped toenails as biological material [68]. However, recent reviews have suggested that toenails were likely inadequate biological material for trace element assessment [69]. A similar concern regarding using toenails was made years ago by Vinceti et al. (2007) when assessing the relationship between Cd content and prostate cancer in humans [70]. Furthermore, several recent studies tend to consider agent content at cancer sites rather than in other tissues [71,72,73]. Several studies have identified that Ni content in PDAC tissue is a reliable biomarker across different studies [67,74,75], while our results also suggest that Ni could be involved in PDAC development in humans.

Studies examining the impact of exposure to various environmental factors on the development of pancreatic cancer are often limited by the relatively small number of cases of pancreatic cancer and the ability to evaluate a small number of environmental factors in studies [76,77,78]. However, a large clinical case study and follow-up indicated the possibility of an association between PDAC risk and Ni exposure [79]. The incidence of pancreatic cancer has been associated with occupational exposure to Ni in several studies [80,81,82,83] and one meta-analysis [84].

The disruption of bio-element homeostasis may be another potential mechanism of Ni carcinogenicity. Iron (Fe)-cell homeostasis disorders were found in our set of PDAC patients when compared to control tissues through a positive correlation between Ni and Fe pancreatic levels (unpublished data). Ni ions have been found to inhibit the histone class, H3K9 demethylase, whose activity depends on Fe and 2-oxoglutarate and increases H3K9 dimethylation [85]. Ni ions compete with Fe ions, which are physiologically linked to the JmjC domain of dioxygenases, enzymes that have catalytic activity [86]. The same study estimated that since Ni ions have a higher affinity with this site, once Ni ions replace Fe ions in this domain, the Fe ions cannot bind again. Oxidative DNA, lipid, and protein damage are significantly increased in the presence of Fe [87,88]. Considering the above results, elevated Ni levels in the body may cause a disturbance of Fe homeostasis, thereby increasing the concentration of this bio-element; this can be a starting point for a further cascade of events that leads to the development of pancreatic cancer. Another recent study suggested that the role of Ni should be further investigated by considering its possible epigenetic effects [18]. The study implied that Ni could be involved in the malignant transformation of pancreatic cell cultures, but also highlighted the importance of toxicants affecting the cells at the same time as Ni, as the endpoint seemed to depend on the presence of other toxicants, as well as Ni concentration. Hence, the precise molecular mechanisms of the role of Ni in PDAC should be addressed through in vitro investigations.

The goal of this paper was to examine not only the transformation of normal cells into cancerous cells, but also the potential for Ni compounds to promote tumour growth in PDAC cell lines. We chose to investigate the effects of Ni exposure at the cellular/molecular level, with higher concentrations of the toxic metal, or longer durations of exposure. It has been reported that Ni at higher concentrations can halt the cell cycle and stop proliferation. In vivo studies have used extremely high doses, up to 900 mg/kg [89], but these doses far exceed what would be considered the upper limit of human exposure, which is 1 mg/day. Evidence from the in vivo studies suggests that Ni stops cell growth in the G_2_/M phase and induces apoptosis through a p53-regulated pathway [89,90]. Other data suggest that Ni-mediated anti-tumour characteristics are mediated by a reduction in angiogenesis, reducing the vasculisation of tumours [91]. A growing body of evidence suggests that Ni and Ni-containing compounds are carcinogenic, a fact recognized by international organisations [13]. The toxicity and carcinogenicity of Ni is supported by our previous data [10,11,18,60], as well as other reports using different cell-line models representing different cancers [92,93,94,95]. The similarity between toxic mechanisms and our work in PDAC cell lines suggests that Ni may work through the Akt pathway, which is independent of FOXO1 activation [92,94]. Using a small-cell carcinoma cell model, it was evident that Ni-induces the activation of VEGF, increasing angiogenesis and the vasculation of the tumour. The activation of VEGF involved Akt, but not FOXO1 [93]. Ni toxicity appears to partially depend on the cell line. Comparing mouse embryo fibroblasts (BALB/3T3) and hepatic tumour cells (HepG2), HepG2 cells appear to have greater sensitivity to the effects of nickel chloride up to 1 mM [95].

Our long-term goal is to study the entire continuum of Ni-induced toxicity. HPNE cells represent ‘normal’ or ‘control’ cells, and we were able to determine the toxicity of NiCl_2_ and Ni-acetate as well as cellular/molecular changes following exposure to Ni. The tumour cell lines vary according to the mutations that are characterized. For example, all cell lines express a *TP53* mutation. Only AsPC-1 cells express the *FBXW7* mutation. Panc-10.05 exhibits the fewest mutations (*TP53* and *KRAS*), but this cell line appears to be the most sensitive to the effects of Ni. Panc-1 and BxPC-3 cells express the same mutations in *TP53*, *CDKN2A*, *MAP2K4* and *SMAD4*. MiaPaCa-2 cells exhibit a deletion of *CDKN2A* and a mutation in *KRAS*, and *TP53*. One question we addressed was whether these deletions have a role in Ni-mediated toxicity. Not only could Ni result in toxic effects and transform a normal, control cell into a cancerous cell, but it may also accelerate tumour growth in tumour cells. By performing LC50 analysis, we were able to visualise any growth/viability changes, even at low (<1 μM) concentrations. We did not observe any effect that would suggest Ni-mediated increase in cell growth.

Lethality curves revealed LC50 values for NiCl_2_ ranging from 196 μM to over 5000 μM, depending on the cell line. Ni-acetate increased (leftward shift of the curve) the lethality in most cell lines, resulting in an LC50 range of 83–1,126 μM, suggesting the increased lethality of the organic Ni form. The values we report are similar in magnitude to those reported in β-cells exposed to NiSO_4_ [96]. We did consider that acetate may change the pH of the media by conversion to acetic acid. The media did contain phenol, and there were no significant changes in the pH of the media, suggesting that pH was not the cause. Furthermore, it may be possible that acetate itself, being a cellular metabolite, has an effect. We have examined the literature and found nothing that would support this hypothesis. In studies examining carcinoma cell lines, the use of acetate can be toxic or promote apoptosis, but only at very high concentrations. In oligodendroglioma cell lines, investigators used 12 mM and 36 mM concentrations of sodium acetate and observed 93+% viability at 12 mM. Our maximum concentration was 10 mM of nickel acetate, suggesting that a reduction in viability was not due to the acetate [97]. Another study found that concentrations of sodium acetate that are comparable to the cellular concentrations generated by complete glyceryl triacetate catalysis promoted growth arrest over an exposure time of 24 h. In HCT-15 and RKO cell lines (colorectal carcinoma), 10 mM acetate reduced viability by less than 10%. Other studies by the same investigators used 70 and 140 mM sodium acetate before significant reductions in viability were observed. An increase in apoptosis, but not necrosis, was reported after 48 h exposure to sodium acetate [98]. In cultured human gastric adenocarcinoma epithelial cells, incubation with up to 12.5 mM sodium acetate for 72 h increased cell viability and proliferation, contrary to what would be expected. The increase in viability and proliferation was concentration-dependent. Concentrations of sodium acetate greater than 12.5 mM inhibited cell growth in a dose-dependent manner by up to 50%. [99].

The most prevalent difference in protein expression/activity was between cell lines. NiCl_2_ increased the expression of phospho-Akt 146% in the BxPC-3 and 83% in the Panc-10.05 cell lines. Ni-acetate increased phospho-Akt expression by 77% in the Panc-10.05 cell line. Both NiCl_2_ and Ni-acetate reduced the expression of FOXO1 in the Panc-10.05 cells by 51% and 19%, respectively. The PTEN expression was elevated by 15% in the Panc-10.05 cells after NiCl_2_ exposure. Exposure to NiCl_2_ or Ni-acetate resulted in caspase 3/7 activity trending downward, with only the HPNE cells demonstrating a significant reduction (43%) in the Ni-acetate group. Collectively, there were minimal effects of either NiCl_2_ or Ni-acetate after 48 h exposure on protein expression or activity. It appears that the Panc-10.05 cell line was most affected by the Ni compounds and in particular, the expression of phospho-Akt and PTEN.

Chickens fed 300–900 mg/kg NiCl_2_ for 14 days demonstrated a generalised increase in hepatic apoptosis with elevated expression of caspase 3, 8, and 9, PARP, and p53, all of which were time- and dose-dependent [90]. The lower dose effects were small after 14 days, but the conclusion was that NiCl_2_ enhanced apoptosis via mitochondrial, Fas, and endoplasmic-reticulum-dependent pathways. Similar results were observed in chicken kidneys receiving the same dosing regimen as the hepatic studies [100]. Similar activation of caspase 3, 7, and 9, as well as increased PARP degradation, was reported in β-cells [96]. The studies by Wu et al. (2011) focused solely on protein content and not activity. In a mouse model, the administration of 7.5, 15, or 30 mg/kg NiCl_2_ resulted in a 70% reduction in the ratio of p-AKT/AKT and a reduction of nearly 40% in mRNA expression in the kidney. The authors concluded that NiCl_2_-induced autophagy took place via the PI3K/AKT/mTOR pathway by reducing the production and activation of AKT (p-AKT), resulting in mTOR inhibition [96]. Our data demonstrated a significant elevation in p-AKT in HPNE control cells, suggesting an increased activation downstream, potentially activating mTOR. Two of the four tumour lines exhibited effects associated with Ni exposure, with both the BxPC-3 and Panc-10.05 cells showing increased p-AKT content after exposure to NiCl_2_, but with no effect of Ni-acetate. A transformed cell line was used to examine the upstream control of AKT via PTEN. Ni was included in the media for two months to transform the cell line, before different cell growth and apoptotic markers were examined. The transformed cells exhibited a significantly higher expression of p-AKT, with reductions in GSK3β and free radical formation [94]. The elevated levels of p-AKT were not due to changes in PTEN regulation (no change in content), but likely due to the PI3K activation of AKT.

Interestingly, in one study, although it was expected that GSK3β would be upregulated and that Wnt/β-catenin would be downregulated, there was no change in β-catenin content or activity. The authors also state that no changes in p53 were observed [101]. These slight differences compared with our study could be due to the different cell lines utilised, and most of the changes observed by the authors were with exposure times greater than 48 h, which was our duration of exposure. Shorter exposures using NiSO_4_ in HepG2 cells resulted in a biphasic AKT response, with an initial increased expression, followed by reduced expression with increasing time [92]. The biphasic effects of Ni on the phosphorylation of AKT were also observed in a different cell model; these effects peaked after about 15 h and began to decline after 24 h [93]. Eckers et al. (2009) also reported downstream changes in the levels of phosphorylation of FOXO1, but not in FOXO1 expression, after 5 min of exposure, up to 60 min [92]. In vivo studies administered Ni over the course of 2 weeks, and up to 6 weeks [89]. The longer cell exposure times to Ni could underscore some of the differences observed in protein expression. The involvement of the AKT/FOXO system is necessary for normal apoptotic function at the cellular level. In our current study, only the Panc-10.05 cells demonstrated a significant reduction in FOXO1 expression. Reduced FOXO1 would remove the inhibition exerted on the Wnt/β-catenin system.

Recent studies have mentioned that specific miR expression profiles could be not only used as promising non-invasive diagnostic biomarkers [101], but also that several miRs play a significant role in metal carcinogen-induced cell malignant transformation and tumorigenesis [102]. Nickel can interfere with miR networks to degrade mRNA or block protein synthesis [103]. Specifically, a previous study indicated that NiCl_2_ exposure could induce the upregulation of miR-210 with the stabilization of the HIF-1α protein, which leads to alterations in metabolism [104,105]. Furthermore, in a recent review, it was mentioned that Ni exposure can cause aberrations in the expression levels of several miRs, which are thought to play a crucial role in tumour growth, cell transformation, and angiogenesis [106]. Several studies have also suggested that Ni exposure can induce the expression levels of miR-152, miR-203, miR-4417, miR-222, and miR-210 [107]. In our current study, we found that the expression of miRs were considerably dysregulated in PDAC cell lines treated with NiCl_2_ compared to corresponding control cells. The miR-221 and miR-155 expression levels were significantly upregulated in the Panc-1 and MiaPaca-2 PDAC cell lines treated with NiCl_2_, while miR-126 was considerably downregulated.

Upregulation of miR-221 expression levels has previously been related with migration, metastasis, and uncontrolled proliferation of PDAC cells [63]. In addition, overexpression of miR-221 can have as an outcome the loss of expression of cyclin-dependent kinases (CDKNs), which is linked to liver fibrosis and unfavourable prognosis of PDAC [107]. It has been recently suggested that the oncogenic effect of miR-221 in PDAC is due to the decrease of PTEN, PUMA, p27 (kip1) and p57 (kip2), which can promote proliferation, inhibit apoptosis and increase the invasive manner of cancer cells [108]. Importantly, when examining the relative expression levels of the selected miRs in our recent study, miR-221 was significantly upregulated in PDAC cells that have been treated with NiCl2 in comparison to the non-treated/control PDAC cells. Furthermore, previous reports have also shown the overexpression of miR-155 during PDAC pathogenesis [52], which is linked to poor prognosis [108]. In our current study, we showed that the expression levels of miR-155 were significantly increased, when PDAC cells were exposed to NiCl2 compared with the control Panc-1 and MiaPaCa-2 cells. A recent study has also mentioned that numerous exosomal miRs such as miR-155, could be potential biomarkers for the early diagnosis of this malignancy [109], while further studies have shown that miR-155 is linked to clinical stage and lymph node metastasis in PDAC patients [110,111,112].

miR-126 downregulation has been associated with the clinical stage, lymph node metastasis, and the unfavourable overall survival of PDAC patients [113]. Several signalling pathways that miR-126 can moderate during PDAC development are Ras/extracellular receptor kinase (ERK), phosphatidylinositol 3-kinase (PI3K)/Ak strain transforming (AKT)/mammalian target of rapamycin (mTOR), A disintegrin and metalloproteinase 9 (ADAM9)/epidermal growth factor receptor (EGFR)/AKT, Ras homolog family member A (RhoA)/Rho-associated kinase (ROCK) and Wnt/β-catenin [114]. Specifically, these pathways are responsible for angiogenesis, cell proliferation, vascular integrity, migration, and invasion in PDAC [115]. The findings of our current study indicated that the expression levels of miR-126 were significantly downregulated in both Panc-1 and MiaPaCa-1 cells following NiCl2 exposure related to the expression levels of control PDAC cells. Therefore, our recent study promotes some pioneer insights into the correlation between NiCl2 exposure and expression levels of oncogenic and tumour suppressor miRs during PDAC pathogenesis.

In summary, our findings suggest novel roles of NiCl2 on PDAC and specifically highlight that Ni plays a crucial role during PDAC development and underscores the role of miRs in response to NiCl2 during PDAC pathogenesis. Our results have also helped indicate the LC50 values for NiCl2 and Ni-acetate in PDAC cell lines and have provided some of the first information regarding the changes in protein expression in PDAC cells after 48 h exposure.

## 5. Conclusions

Prompted by the interesting results obtained in human pancreatic tissue, this is the first study to examine NiCl_2_ treatment in PDAC cells. Using 48 h exposure times and PDAC cell lines, we established LC50 values for both NiCl_2_ and Ni-acetate. These LC50 values will be used in future research, in which we will use subtoxic concentrations for extended periods, while examining cellular and molecular changes. Most of the observed differences in protein expression were cell-line-dependent, but there were instances, most notably in the BxPC-3 and Panc-10.05 cells lines, where NiCl_2_ and Ni-acetate exposure resulted in expression changes. One shortcoming of the present study is that, while investigating the immediate toxic effects following Ni exposure, the 48-h time course of exposure may be too short. The 48-h time course was based on previous studies with other toxic metals, and may have been too short to determine changes in protein expression, which would take longer to manifest. Additional studies would be needed for a longer duration of Ni exposure to determine more robust changes in protein expression. There are multiple miRs involved in regulating cellular function, and miR-21 and miR-27a are reported to regulate the activity of AKT [19,20,21,22]. In this study, Panc-1 and MiaPaCa-2 were treated with NiCl_2_, and the results suggested that the expression levels of the selected miRs were significantly disrupted, including the increasing pro-oncogenic properties of miR-221 and miR-155 and the decreasing anti-oncogenic capabilities of miR-126. Our results report novel regulatory roles of NiCl_2_ in PDAC, and especially underline the role of NiCl_2_ exposure in the alteration of miR expression levels during PDAC pathogenesis. With emerging attention to miRs and the development of new approaches for understanding miRs, several new mechanisms that lead to PDAC initiation and progression, such as NiCl_2_ exposure, were noted. Conclusively, our study provides pioneering insights into the pathogenesis and mechanisms of PDAC, promoting effective prognostic approaches.

## Figures and Tables

**Table 1:** Summary of LC50 values for NiCl_2_ and Ni-acetate in PDAC cell lines.

	Metals (µM ± S.D.)	
Cell Type	NiCl_2_	Ni-Acetate
HPNE	319.8 ± 24.1	167.3 ± 28.7
AsPC-1	196.3 ± 26.9	81.3 ± 6.1
Panc-1	1068 ± 69.3 ^#^	1128 ± 101.3 ^†,^*
BxPC-3	953.5 ± 132.5 *	290.6 ± 38.8 ^#^
Panc-10.05	>5000 ^†^	183.5 ± 22.8
MiaPaCa-2	4209 ± 875 ^†^	119.3 ± 33.1 ^a^

^†^*p* < 0.0001 Compared to HPNE and AsPC-1; ^#^ *p* < 0.01 compared to AsPC-1; * *p* < 0.05 compared to AsPC-1 for NiCl_2_ exposure. For Ni-acetate exposure, ^†^ *p* < 0.001 compared to HPNE and AsPC-1; ^#^ compared to AsPC-1; ^a^ *p* < 0.001 compared to Panc-1; * *p* < 0.05 compared to Panc-10.05. Data are expressed as the mean ±S.D. of 8 experiments run in duplicate. Data analysis consisted of Kruskal-Wallis analysis followed by post-hoc analyses using Dunn’s test for multiple comparisons.

## Data Availability

The data is available on request.

## Abbreviations

ADAM9	A disintegrin and metalloproteinase 9
AKT	Ak strain transforming
Bad	Bcl-2-associated death promoter
CD	Cadmium
CDKNs	Cyclin-dependent kinases
CDKN2A	Cyclin-dependent kinase inhibitor 2A
DMSO	Dimethylsulfoxide
EGFR	Epidermal growth factor receptor
ERK	Extracellular receptor kinase
FBS	Foetal bovine serum
FOXO-1	Forkhead box protein O1
GSK3β	Glycogen Synthase Kinase 3 Beta
HER2	Human epidermal growth factor receptor 2
HIF-1	Hypoxia-inducible-factor-1
HPNE	Human pancreatic nestin-expressing
HRP	Horseradish peroxidase
IARC	International Agency for Research on Cancer
IKK-ɑ	IkappaB kinase
K-RAS	Kirsten rat sarcoma viral homolog
LC50	Lethal concentration 50%: concentration that is lethal to half the population of cells
MDM2	Mouse double minute 2 homolog or E3 ubiquitin-protein ligase
miRs/miRNAs	microRNAs
mRNAs	Messenger RNAs
mTOR	mechanistic target of rapamycin
Ni	Nickel
Ni-acetate	Nickel acetate
NiCl_2_	Nickel chloride
PanIN	Pancreatic intraepithelial neoplasia
PARP	Poly (ADP-ribose) polymerase
PBS	Phosphate-buffered saline
PDAC	Pancreatic ductal adenocarcinoma
phospho-AKT	Phosphorylated protein kinase B (PKB)
PI3K	Phosphatidylinositol 3-kinase
PIP2	Phosphatidylinositol 4,5-bisphosphate
PIP3	Phosphatidylinositol 3,4,5-trisphosphate
PTEN	Phosphatase and tensin homolog
RhoA	Ras homolog family member A
ROCK	Rho-associated kinase
SD	Standard deviation
tp53	Tumour protein 53
UTRs	Untranslated regions
